# Supplementary material for: Accurate statistical methods to cover the aspects of the increase in the incidence of kidney failure: A survey study in Ha’il -Saudi Arabia
Source: PLoS One. 2024 Aug 28;19(8):e0309226. doi: 10.1371/journal.pone.0309226 (PMC11357112; doi:10.1371/journal.pone.0309226)
Supplement: S1 Data — (PDF) [file pone.0309226.s001.pdf]

Lack of drinl	taking r	diabete	hyperte	heart di	lack of (	obesity	recurrin	poor die	smoking
1	-1	1	1	1	-1	1	1	1	1
1	1	-1	-1	-1	1	-1	-1	1	1
1	-1	-1	-1	-1	1	1	1	1	1
1	1	-1	-1	-1	-1	-1	-1	-1	1
1	1	1	1	1	1	1	1	1	1
1	-1	-1	-1	-1	-1	-1	-1	1	-1
1	-1	-1	-1	-1	1	1	1	1	1
-1	-1	-1	-1	-1	-1	-1	1	1	-1
1	1	-1	-1	-1	1	-1	1	1	1
1	1	1	1	1	1	1	1	1	1
1	1	1	1	1	1	-1	-1	1	1
1	1	1	1	1	1	1	1	1	1
1	-1	1	1	1	-1	-1	1	1	1
1	-1	-1	-1	-1	1	1	1	-1	1
-1	1	1	-1	-1	1	-1	1	1	-1
1	1	1	1	1	1	-1	-1	-1	1
1	-1	-1	1	-1	1	-1	1	-1	-1
1	-1	1	1	-1	-1	1	1	-1	1
1	1	-1	-1	-1	1	1	1	-1	1
1	1	1	1	-1	-1	-1	1	1	1
1	1	1	1	1	1	1	1	1	1
1	1	1	1	1	1	1	1	1	1
1	1	1	1	1	1	1	1	1	1
1	1	1	1	1	1	1	1	1	1
-1	-1	-1	-1	-1	1	1	-1	1	-1
1	1	1	-1	1	-1	1	1	-1	1
1	1	-1	-1	-1	-1	1	1	1	1
1	1	1	1	-1	-1	-1	1	1	1
-1	1	-1	-1	-1	1	1	1	1	1
1	1	-1	-1	-1	-1	1	-1	1	1
1	1	1	1	-1	1	-1	-1	1	-1
1	1	-1	-1	-1	-1	-1	-1	1	1
1	1	-1	-1	1	1	-1	1	-1	1
1	1	1	-1	-1	1	1	-1	1	1
1	1	1	1	-1	-1	-1	1	1	1
1	1	-1	-1	1	1	1	1	1	1
1	-1	-1	-1	-1	-1	-1	1	1	-1
1	1	-1	-1	-1	1	1	1	1	1
1	1	1	1	-1	1	1	1	1	1
1	1	-1	-1	1	-1	-1	-1	1	1
1	1	-1	-1	-1	1	-1	1	1	-1
1	1	1	-1	-1	1	1	1	1	1
1	1	1	1	1	1	1	1	1	1

1	1	-1	-1	-1	1	-1	-1	1	-1
1	1	1	1	-1	-1	-1	1	1	1
1	-1	-1	-1	-1	1	1	1	1	-1
1	1	1	1	1	1	1	1	1	1
1	-1	-1	-1	-1	1	1	1	1	1
1	-1	1	1	-1	1	-1	1	-1	1
1	1	1	1	-1	-1	-1	-1	-1	1
1	1	-1	-1	-1	-1	-1	-1	1	1
1	1	-1	1	-1	1	1	1	1	1
1	1	1	1	1	1	-1	1	1	1
1	1	-1	-1	-1	1	-1	-1	1	1
1	1	1	1	1	1	1	1	1	1
1	1	1	-1	-1	1	1	1	1	1
1	-1	-1	-1	-1	-1	1	1	1	1
1	1	-1	-1	-1	-1	1	-1	-1	1
1	1	1	1	-1	-1	-1	1	1	-1
1	1	-1	-1	-1	1	1	1	1	1
1	1	1	-1	-1	1	1	1	1	-1
1	1	-1	-1	-1	-1	1	1	1	1
1	1	-1	-1	-1	1	1	1	1	1
1	1	1	1	1	1	1	1	1	1
1	1	1	1	1	-1	1	1	1	1
1	1	1	1	1	1	-1	-1	1	1
1	1	-1	-1	-1	-1	-1	1	-1	-1
1	1	-1	-1	-1	-1	1	1	-1	-1
1	1	-1	-1	-1	-1	-1	1	1	1
-1	-1	1	1	1	1	-1	-1	1	1
1	1	1	1	-1	-1	-1	1	1	1
1	1	-1	-1	-1	1	-1	-1	1	1
1	1	-1	-1	-1	1	1	-1	1	1
-1	-1	-1	-1	-1	-1	-1	-1	-1	-1
1	1	1	1	1	1	1	1	1	1
1	-1	1	1	1	1	1	1	1	1
1	1	1	-1	-1	1	1	1	1	1
1	1	1	1	1	1	1	1	1	1
-1	-1	-1	-1	-1	-1	-1	-1	-1	-1
1	1	-1	-1	-1	1	1	1	1	1
1	1	-1	1	-1	1	1	1	1	-1
1	1	-1	-1	-1	-1	-1	-1	1	1
1	1	1	1	-1	1	1	1	1	1
1	-1	-1	-1	-1	1	1	1	1	1
1	1	-1	-1	-1	1	1	1	1	1
1	-1	-1	-1	1	1	1	1	1	1
1	1	1	-1	-1	1	1	1	-1	-1

1	1	-1	-1	-1	-1	1	1	1	1
1	1	-1	-1	-1	1	1	1	1	-1
1	1	-1	-1	-1	-1	-1	1	1	1
1	1	1	1	1	1	1	1	1	1
1	1	-1	-1	-1	-1	-1	1	1	-1
1	-1	1	1	-1	-1	-1	1	1	1
-1	1	-1	-1	-1	-1	-1	1	-1	1
1	1	-1	-1	-1	-1	-1	-1	1	-1
1	1	-1	-1	-1	1	1	1	1	1
1	1	1	-1	-1	-1	1	1	1	1
1	1	-1	1	-1	1	-1	1	1	1
1	1	-1	1	-1	1	1	1	1	-1
-1	1	1	1	-1	-1	1	1	1	1
1	1	-1	-1	-1	-1	-1	1	1	1
1	-1	-1	-1	-1	-1	-1	1	-1	1
1	1	1	-1	-1	-1	-1	1	1	1
1	-1	1	-1	-1	1	1	-1	1	1
1	1	-1	-1	-1	1	1	-1	1	1
1	1	1	1	-1	-1	-1	1	-1	-1
1	1	1	1	1	1	1	1	1	1
1	1	-1	-1	-1	-1	-1	1	1	1
1	1	-1	-1	1	1	1	1	1	1
1	1	1	1	1	1	1	1	1	1
1	1	-1	1	1	-1	-1	-1	-1	-1
-1	1	-1	-1	1	-1	-1	1	1	1
1	1	1	1	1	1	1	1	1	1
1	1	-1	-1	-1	1	1	1	1	1
1	1	-1	-1	-1	-1	-1	-1	-1	-1
1	1	1	1	1	-1	1	1	1	1
1	1	1	-1	-1	1	1	-1	1	1
1	1	1	1	-1	-1	-1	1	-1	1
1	1	1	1	1	-1	1	-1	-1	-1
-1	1	-1	-1	-1	-1	1	1	1	1
1	-1	1	-1	1	-1	1	-1	1	-1
1	1	1	-1	-1	1	1	1	1	1
1	1	1	1	1	1	1	-1	1	1
1	1	1	-1	-1	-1	-1	1	-1	-1
-1	1	1	1	1	-1	1	1	1	-1
-1	1	1	1	1	-1	1	1	1	1
1	-1	-1	-1	1	-1	-1	-1	1	1
-1	1	1	1	1	-1	1	1	1	1
1	1	1	1	1	1	-1	1	1	1
1	1	1	1	1	1	1	-1	-1	-1
1	1	1	1	1	1	-1	-1	1	1

-1	1	1	1	1	-1	1	-1	1	1
1	1	1	1	1	-1	1	1	1	1
-1	1	1	1	1	1	1	-1	1	1
1	-1	-1	-1	-1	1	-1	1	1	-1
1	1	1	-1	-1	1	1	1	1	1
1	-1	-1	-1	1	1	1	1	1	1
1	1	1	1	1	-1	1	1	1	1
1	1	-1	-1	-1	1	1	-1	1	1
1	1	-1	1	1	-1	-1	1	1	1
-1	-1	1	1	1	-1	-1	1	-1	-1
1	-1	1	-1	-1	1	1	-1	1	1
1	1	1	1	1	1	1	-1	1	1
1	-1	1	1	1	1	1	1	1	1
1	1	-1	-1	-1	1	1	-1	1	1
1	1	1	-1	-1	1	1	1	1	1
1	1	-1	-1	-1	1	1	1	1	1
1	-1	1	-1	-1	1	1	-1	1	1
1	1	1	-1	-1	1	1	1	1	-1
1	-1	1	1	-1	1	1	1	-1	-1
1	-1	1	1	-1	1	1	1	1	1
1	1	1	-1	-1	1	1	-1	1	-1
1	1	1	1	1	1	1	1	1	1
1	1	1	-1	-1	1	1	-1	1	1
1	1	1	1	1	1	1	1	1	1
1	1	1	-1	-1	1	1	1	1	1
1	1	1	1	1	1	1	1	1	1
1	1	1	1	1	-1	-1	-1	1	1
-1	-1	-1	-1	-1	-1	-1	-1	-1	-1
1	1	1	-1	-1	-1	1	1	1	1
1	1	-1	-1	1	1	1	-1	1	1
1	1	1	1	-1	-1	1	1	-1	-1
1	1	-1	1	-1	-1	-1	-1	1	-1
1	-1	-1	-1	-1	1	-1	-1	1	-1
1	-1	-1	-1	-1	1	1	1	1	1
1	-1	-1	-1	-1	1	1	1	1	1
1	1	1	1	1	1	1	1	1	1
1	-1	-1	-1	-1	1	1	-1	-1	-1
1	-1	-1	-1	-1	1	-1	-1	-1	-1
1	1	1	-1	1	-1	-1	-1	-1	-1
1	1	1	-1	-1	1	1	1	1	1
1	1	1	1	1	1	1	1	1	1
-1	1	1	1	1	-1	1	1	-1	1
1	1	1	-1	1	1	1	1	1	1
1	1	1	-1	-1	1	1	-1	1	1

1	1	1	1	1	-1	1	1	-1	1
1	1	1	1	1	-1	1	1	1	1
1	1	1	1	1	1	1	1	1	1
1	1	1	1	1	1	1	1	1	1
1	1	1	1	1	1	1	-1	1	1
1	1	1	1	1	1	1	1	1	1
1	1	1	1	1	1	1	1	1	1
1	-1	-1	-1	-1	-1	-1	-1	-1	-1
1	1	1	1	1	-1	-1	1	1	1
1	-1	-1	-1	-1	-1	-1	-1	-1	-1
1	1	-1	-1	-1	-1	-1	1	-1	1
1	1	1	1	1	1	-1	-1	-1	1
1	1	1	1	1	1	1	1	1	1
1	1	-1	-1	-1	1	-1	-1	1	-1
1	1	1	1	1	1	1	1	1	1
1	-1	1	1	-1	1	1	1	1	-1
1	-1	1	-1	1	1	1	-1	1	1
1	1	1	-1	-1	-1	-1	-1	-1	1
1	1	1	1	1	1	1	1	1	1
1	1	1	-1	-1	1	1	1	1	1
1	-1	-1	-1	-1	-1	-1	1	-1	1
1	1	1	1	1	1	1	1	1	-1
1	1	-1	-1	-1	-1	1	1	1	1
1	1	-1	-1	-1	1	1	1	1	1
1	1	1	1	-1	-1	-1	1	1	1
-1	1	1	1	-1	-1	-1	-1	-1	1
1	1	-1	-1	-1	1	-1	1	1	1
1	1	-1	-1	-1	-1	-1	1	-1	1
1	1	1	1	1	1	1	1	-1	1
1	1	1	1	1	1	1	1	1	1
1	1	-1	-1	-1	1	1	1	1	1
-1	-1	-1	-1	-1	1	1	-1	1	-1
1	-1	-1	-1	-1	-1	-1	-1	1	1
1	1	1	-1	-1	1	1	-1	1	1
1	1	1	1	-1	-1	-1	1	-1	1
1	-1	1	1	1	-1	-1	1	1	1
1	1	-1	-1	-1	1	1	1	1	1
1	1	1	1	1	1	1	1	1	1
1	-1	-1	-1	-1	-1	-1	-1	1	-1
1	1	1	1	-1	-1	-1	1	1	-1
1	1	1	1	-1	1	1	1	1	1
1	1	1	-1	-1	-1	-1	1	-1	-1
1	1	1	1	-1	-1	1	1	-1	1
1	-1	-1	-1	1	-1	1	1	1	1

1	1	1	1	1	1	1	1	1	1
-1	1	-1	-1	-1	-1	-1	-1	-1	-1
1	1	-1	-1	-1	-1	1	1	1	1
1	1	-1	-1	-1	1	1	1	1	1
-1	-1	1	1	1	1	1	1	1	-1
1	1	1	-1	-1	1	-1	1	1	-1
1	1	-1	-1	1	-1	1	1	1	1
1	1	1	1	1	-1	-1	1	-1	1
1	-1	-1	-1	-1	-1	-1	-1	-1	1
1	1	-1	-1	-1	1	1	1	1	1
1	1	-1	1	1	1	1	-1	1	1
1	-1	-1	-1	-1	-1	-1	1	1	1
1	1	1	-1	-1	1	1	1	1	1
1	1	-1	-1	1	1	-1	-1	1	1
1	1	1	1	1	1	1	1	1	1
-1	-1	-1	-1	-1	-1	-1	-1	1	-1
1	1	1	1	1	1	1	-1	-1	-1
1	1	-1	-1	-1	-1	-1	-1	-1	1
1	1	1	1	-1	-1	1	1	1	1
1	1	1	-1	-1	1	1	1	1	1
1	1	-1	1	1	1	1	1	1	1
1	-1	-1	-1	-1	1	1	1	1	1
1	1	-1	-1	-1	1	-1	-1	1	1
-1	-1	-1	-1	-1	-1	1	-1	1	-1
1	-1	1	1	1	1	1	1	1	1
1	1	1	-1	-1	1	1	1	1	1
1	1	-1	-1	-1	1	1	1	1	1
1	1	1	-1	-1	-1	-1	-1	1	1
1	1	-1	-1	1	-1	-1	-1	1	1
1	1	-1	1	-1	-1	-1	1	1	1
1	1	-1	-1	-1	-1	-1	1	1	1
1	1	1	1	1	-1	-1	1	-1	1
1	1	-1	1	-1	-1	-1	-1	1	1
1	-1	-1	-1	-1	-1	-1	1	1	1
1	1	-1	-1	-1	-1	-1	1	1	1
-1	-1	-1	-1	-1	-1	-1	-1	-1	-1
1	1	1	1	1	1	1	1	1	1
1	1	1	1	1	1	1	1	1	1
1	1	-1	-1	-1	-1	-1	1	-1	1
1	1	1	1	1	1	1	1	1	1
1	1	1	1	1	-1	1	-1	1	1
1	1	1	-1	-1	-1	-1	1	1	1
1	1	-1	-1	-1	1	1	1	1	1
1	1	-1	-1	-1	1	1	1	1	1

1	1	-1	-1	1	1	1	1	1	1
-1	-1	1	1	1	-1	-1	-1	-1	-1
1	1	-1	1	-1	1	1	1	1	1
1	1	-1	-1	-1	1	1	1	1	-1
1	-1	-1	-1	-1	-1	-1	1	1	-1
1	1	1	1	-1	-1	1	1	1	1
-1	1	-1	-1	1	-1	1	1	1	1
1	1	-1	1	-1	-1	-1	1	1	1
1	1	-1	-1	-1	1	1	-1	1	1
1	1	1	1	1	1	1	-1	-1	1
1	1	-1	-1	-1	-1	-1	1	-1	-1
1	1	1	1	-1	1	1	1	1	1
1	1	1	1	1	1	1	-1	1	1
1	1	-1	-1	-1	1	1	1	1	1
1	-1	-1	-1	-1	1	-1	-1	-1	-1
1	-1	1	1	1	1	1	1	1	1
1	1	-1	1	1	-1	-1	1	1	1
1	1	-1	-1	-1	-1	-1	1	1	1
1	1	-1	-1	-1	-1	-1	-1	1	1
1	1	-1	-1	-1	-1	1	1	1	1
1	1	1	1	-1	-1	-1	1	1	-1
1	1	-1	-1	-1	-1	-1	1	1	1
-1	1	-1	-1	-1	-1	1	1	1	1
1	-1	1	1	-1	1	1	1	1	1
1	1	-1	-1	-1	-1	-1	1	1	1
1	1	-1	-1	-1	1	1	-1	1	1
1	1	1	1	1	1	1	1	1	1
-1	1	1	1	1	1	-1	1	-1	-1
1	1	1	-1	-1	1	1	-1	1	1
1	1	1	-1	-1	-1	1	-1	1	1
1	1	1	1	1	-1	1	1	-1	1
1	1	-1	1	1	1	1	1	1	-1
-1	-1	1	-1	-1	-1	-1	1	-1	-1
1	1	-1	-1	1	-1	1	1	1	1
1	1	1	1	-1	1	1	-1	1	1
1	1	-1	-1	-1	1	1	1	1	1
1	1	-1	-1	-1	1	1	1	1	1
1	1	1	1	1	1	1	1	1	1
1	-1	-1	-1	-1	1	-1	1	1	1
1	1	1	1	-1	1	1	-1	1	1
1	1	1	1	1	1	1	1	-1	1
1	-1	-1	-1	1	-1	1	-1	1	1
1	1	1	-1	-1	1	1	1	1	1
1	1	1	1	1	1	1	1	1	1

-1	-1	-1	-1	-1	-1	-1	-1	-1	-1
1	1	1	1	1	1	1	-1	-1	1
-1	-1	-1	-1	-1	-1	-1	-1	-1	-1
1	1	-1	1	-1	-1	-1	1	1	-1
1	1	1	1	1	1	1	1	1	1
1	1	-1	-1	-1	-1	-1	-1	-1	1
1	1	1	1	1	-1	1	1	-1	1
1	1	-1	-1	-1	1	1	1	1	-1
1	1	-1	1	1	1	-1	-1	1	1
1	1	-1	-1	-1	1	1	1	1	-1
1	1	1	1	1	1	1	1	1	1
-1	-1	1	-1	1	1	-1	-1	1	1
1	-1	-1	1	-1	-1	1	-1	1	-1
1	1	1	-1	-1	1	1	-1	1	1
1	1	-1	-1	1	1	1	-1	1	1
1	1	-1	-1	-1	1	-1	-1	1	1
1	1	-1	-1	-1	1	-1	1	1	-1
1	1	-1	-1	-1	-1	-1	1	1	1
1	1	1	-1	-1	1	1	1	-1	-1
1	1	-1	1	1	1	1	1	1	-1
1	1	1	1	1	1	1	1	1	1
1	1	-1	-1	-1	1	-1	1	1	1
1	1	1	1	1	1	1	1	1	1
1	1	1	1	1	1	1	1	1	1
1	1	1	1	-1	1	1	1	1	1
-1	1	-1	1	1	-1	-1	1	1	1
-1	-1	1	1	-1	-1	-1	-1	-1	-1
1	1	1	1	1	1	1	1	1	1
1	1	1	1	1	1	1	1	1	1
1	1	1	1	1	1	1	1	1	1
1	1	1	1	1	1	1	1	1	1
1	1	1	1	1	-1	-1	-1	-1	1
-1	-1	1	1	-1	-1	-1	-1	-1	-1
1	1	1	1	1	-1	-1	1	1	1
-1	1	1	1	-1	-1	-1	1	1	1
1	1	1	1	1	1	1	1	1	1
1	-1	-1	-1	-1	1	1	-1	1	1
1	1	1	1	1	1	1	1	-1	1
1	1	1	1	1	1	1	1	1	1
1	-1	-1	-1	-1	-1	1	1	-1	-1
-1	1	1	-1	-1	-1	-1	1	-1	-1
1	-1	-1	-1	-1	1	1	-1	1	1
-1	-1	-1	-1	-1	-1	-1	-1	-1	-1
1	1	-1	-1	-1	-1	-1	-1	1	1

1	1	-1	-1	-1	-1	-1	-1	1	1
1	1	1	1	1	1	1	1	1	1
1	1	1	1	1	1	1	1	1	1
1	1	1	1	-1	-1	-1	1	-1	1
1	1	1	-1	-1	1	-1	1	1	1
-1	1	1	1	1	1	1	1	1	1
-1	1	1	1	-1	-1	-1	1	-1	1
1	1	1	1	-1	1	1	-1	1	1
1	-1	-1	-1	-1	1	1	1	1	1
1	1	1	1	1	-1	1	1	-1	-1
1	-1	-1	-1	-1	1	1	1	1	1
1	1	-1	-1	-1	1	-1	-1	1	-1
1	1	1	1	1	1	-1	-1	-1	1
1	1	1	1	1	-1	1	-1	1	-1
-1	-1	-1	-1	-1	1	1	1	1	1
1	1	-1	-1	-1	1	1	-1	1	1
1	1	-1	-1	-1	-1	1	1	1	-1
1	-1	1	-1	-1	-1	-1	1	1	1
-1	-1	-1	-1	-1	-1	-1	-1	-1	-1
1	1	1	1	1	1	-1	-1	-1	-1
1	1	-1	-1	-1	1	1	-1	1	1
1	1	1	1	-1	1	1	1	1	1
1	-1	1	1	-1	1	1	1	1	1
1	-1	-1	1	1	1	1	1	1	1
-1	-1	-1	-1	-1	-1	-1	-1	-1	-1
1	1	1	1	1	1	1	1	1	1
1	-1	-1	-1	-1	-1	-1	1	-1	1
1	1	1	1	1	1	1	1	1	1
1	1	1	1	1	1	1	1	1	1
1	1	1	1	1	1	1	1	1	1
1	1	1	-1	-1	-1	-1	1	1	-1
1	1	1	1	1	1	1	-1	-1	1
1	1	1	1	1	-1	-1	1	1	-1
1	1	1	1	1	1	1	1	1	1
1	1	-1	-1	-1	-1	-1	1	1	1
1	1	-1	-1	-1	1	1	1	1	1
1	1	1	1	1	1	1	1	1	1
1	1	1	1	1	1	1	1	1	1
1	1	1	1	1	1	1	1	1	1
1	1	-1	-1	1	1	1	1	1	1
1	1	-1	-1	-1	1	1	1	1	1
1	1	1	1	1	1	1	1	1	1
1	1	-1	-1	-1	-1	-1	1	1	1
1	1	1	-1	-1	1	-1	-1	-1	1

1	1	1	1	1	1	1	1	1	1
1	1	-1	-1	-1	-1	-1	1	-1	-1
1	-1	-1	-1	-1	1	1	-1	1	1
1	-1	-1	-1	-1	1	1	1	1	1
1	-1	-1	-1	-1	1	1	-1	1	1
1	-1	1	1	1	1	-1	1	-1	-1

presenc	Sustain	Direct c	Drinking	Dyes us	Stress a	Excessi	Rheuma	How many case
1	1	1	1	-1	-1	1	-1	2
1	-1	1	1	-1	-1	1	-1	3
1	1	1	1	1	1	1	-1	8
1	1	1	-1	1	1	-1	-1	4
1	-1	1	1	1	-1	1	-1	7
1	-1	1	-1	-1	1	1	-1	2
-1	1	-1	-1	-1	1	1	-1	2
1	-1	1	-1	-1	-1	-1	-1	1
1	-1	1	1	1	-1	1	-1	2
1	1	1	1	1	-1	1	-1	5
1	1	1	1	1	1	1	1	1
1	1	1	1	1	1	1	1	3
-1	-1	-1	1	1	-1	1	-1	2
1	-1	1	-1	-1	-1	1	-1	1
-1	1	-1	1	-1	1	-1	1	10
1	1	1	-1	1	-1	-1	-1	0
-1	-1	-1	-1	-1	-1	1	-1	2
1	-1	1	-1	1	1	-1	1	2
1	-1	1	1	1	-1	1	-1	2
-1	-1	1	1	-1	-1	1	-1	5
1	1	1	1	1	1	1	1	1
1	1	1	1	1	1	1	1	2
1	1	1	1	1	1	1	1	3
-1	-1	1	-1	-1	-1	1	-1	2
-1	-1	-1	-1	-1	-1	-1	-1	2
1	-1	1	1	-1	-1	1	1	0
1	-1	1	1	-1	-1	1	-1	2
1	-1	1	-1	1	-1	-1	-1	1
1	1	1	1	1	1	1	1	2
1	-1	1	-1	-1	-1	1	-1	1
1	1	1	1	-1	-1	1	-1	0
1	-1	1	1	1	-1	1	1	0
1	-1	-1	1	1	-1	1	-1	1
1	-1	1	1	1	1	1	-1	2
1	-1	-1	-1	-1	-1	1	-1	2
1	1	1	1	1	1	1	1	3
-1	-1	-1	-1	-1	1	1	-1	0
1	-1	1	-1	1	-1	1	-1	2
1	-1	1	-1	-1	1	1	-1	1
-1	-1	1	1	-1	-1	-1	-1	0
1	1	1	1	1	1	1	-1	3
1	1	1	1	1	-1	1	-1	1
1	1	1	1	1	1	1	1	18

-1	-1	1	1	-1	-1	1	-1	2
1	1	1	1	1	-1	1	1	2
1	-1	1	-1	-1	-1	1	-1	1
1	1	1	1	1	1	1	1	3
1	-1	1	-1	-1	1	1	-1	4
1	1	1	1	-1	1	1	-1	1
1	1	1	-1	-1	-1	-1	-1	1
1	-1	1	1	-1	-1	1	-1	1
1	-1	1	-1	-1	-1	1	-1	1
1	-1	1	-1	1	-1	1	1	3
1	-1	-1	-1	-1	-1	1	-1	4
1	1	1	1	1	1	1	1	1
1	-1	1	-1	1	-1	1	1	0
1	1	1	1	1	-1	-1	-1	1
1	-1	1	1	-1	-1	1	-1	1
1	1	1	1	1	-1	-1	-1	3
1	1	1	1	1	1	1	-1	3
-1	-1	1	-1	-1	-1	-1	1	2
1	-1	-1	1	-1	-1	1	-1	2
1	1	1	1	1	-1	-1	1	1
1	1	1	1	1	1	1	1	5
1	-1	1	1	-1	1	1	1	3
1	-1	1	-1	1	-1	-1	-1	1
1	-1	1	1	1	-1	-1	-1	1
1	-1	1	1	1	-1	-1	1	1
1	-1	1	1	-1	-1	-1	-1	25
1	-1	-1	-1	1	-1	1	1	1
1	-1	1	-1	1	-1	1	-1	1
-1	1	1	1	-1	-1	-1	-1	0
1	-1	1	1	-1	1	1	-1	9
-1	-1	-1	-1	-1	-1	-1	-1	2
1	1	1	1	1	1	1	1	1
1	1	1	1	1	-1	1	-1	3
1	1	1	1	1	-1	1	-1	0
1	1	1	1	1	1	1	1	1
-1	-1	-1	-1	-1	-1	-1	-1	6
1	1	1	1	1	1	1	-1	0
1	1	1	1	1	1	1	-1	0
1	-1	1	1	-1	-1	1	-1	1
1	-1	1	1	1	-1	1	1	0
1	-1	1	1	-1	1	1	-1	0
1	-1	1	1	-1	-1	-1	-1	1
1	1	1	1	-1	-1	1	-1	2
-1	-1	-1	1	-1	-1	1	-1	0

1	-1	1	1	1	-1	1	-1	1
-1	-1	-1	1	1	-1	1	-1	3
1	1	1	1	1	1	1	-1	0
1	1	1	1	1	1	1	1	5
1	-1	1	-1	-1	-1	1	-1	1
1	1	1	-1	-1	-1	1	-1	2
1	-1	1	-1	-1	-1	-1	-1	0
-1	-1	1	1	-1	-1	-1	-1	0
1	1	1	1	1	1	1	1	0
1	-1	1	1	1	1	1	-1	1
1	-1	1	-1	1	1	1	-1	0
-1	-1	-1	-1	-1	1	1	-1	1
1	1	1	1	1	1	1	-1	2
1	1	1	1	1	-1	1	1	2
1	1	1	-1	1	1	1	-1	2
1	-1	-1	-1	-1	-1	-1	-1	0
-1	-1	-1	-1	-1	-1	1	-1	0
1	1	1	1	1	1	1	1	1
-1	1	1	1	-1	-1	1	-1	5
1	-1	1	-1	-1	1	1	-1	0
1	-1	-1	1	-1	-1	1	-1	7
1	-1	-1	1	1	1	-1	-1	0
1	1	1	1	1	1	1	1	5
-1	1	1	-1	-1	-1	-1	1	2
1	-1	1	-1	-1	-1	-1	-1	3
1	1	1	1	1	1	1	1	0
1	1	1	1	1	-1	1	-1	0
-1	-1	1	-1	-1	-1	1	-1	0
1	-1	-1	1	1	1	1	-1	10
1	-1	1	-1	-1	-1	1	-1	8
-1	-1	-1	-1	-1	-1	1	-1	0
1	-1	1	1	-1	-1	-1	1	3
1	-1	1	1	-1	-1	1	-1	1
1	1	-1	1	1	-1	1	1	5
1	-1	1	1	-1	-1	1	-1	0
1	1	1	-1	1	-1	1	1	0
1	-1	1	-1	-1	-1	1	-1	2
1	1	1	1	1	-1	1	-1	1
1	1	1	1	-1	-1	1	-1	1
1	-1	1	-1	-1	-1	1	-1	0
1	1	1	-1	-1	-1	1	-1	2
1	1	1	1	1	1	1	1	0
-1	-1	1	-1	-1	-1	1	-1	0
-1	-1	1	1	-1	-1	1	-1	0

1	-1	1	-1	-1	-1	1	-1	3
1	1	1	1	1	1	1	1	0
-1	-1	-1	-1	-1	1	1	-1	0
1	-1	1	-1	-1	-1	1	-1	5
1	-1	1	1	-1	-1	-1	-1	1
1	1	1	1	1	1	-1	1	0
1	1	1	1	-1	-1	1	-1	0
1	1	1	-1	-1	1	1	-1	1
1	-1	1	1	1	-1	1	1	2
-1	-1	-1	-1	-1	1	1	-1	0
1	-1	-1	-1	-1	-1	1	-1	2
1	-1	1	1	1	1	1	1	3
1	1	1	-1	1	1	1	1	2
1	-1	1	-1	-1	-1	-1	-1	0
1	-1	1	-1	1	1	1	-1	2
1	-1	1	1	1	-1	1	-1	2
-1	-1	-1	-1	-1	-1	1	-1	2
1	-1	-1	-1	-1	-1	1	-1	4
1	-1	1	-1	-1	-1	1	-1	3
-1	-1	1	1	1	-1	1	1	2
1	-1	1	-1	-1	-1	-1	-1	0
1	-1	1	1	1	1	1	-1	2
1	-1	1	1	1	-1	1	1	2
1	-1	1	-1	-1	-1	1	-1	1
1	1	1	1	1	1	1	1	2
1	1	1	1	1	1	1	1	2
1	-1	1	-1	-1	-1	1	-1	1
-1	-1	-1	-1	-1	-1	-1	-1	2
1	1	-1	1	-1	-1	1	-1	6
-1	-1	-1	1	-1	-1	1	-1	0
1	-1	1	-1	1	1	1	-1	3
-1	-1	1	1	1	-1	1	-1	5
-1	-1	-1	-1	-1	-1	-1	-1	0
-1	-1	1	-1	-1	1	1	1	1
1	-1	1	1	-1	1	1	-1	3
1	-1	1	1	-1	1	1	1	0
-1	-1	-1	-1	-1	-1	1	-1	1
-1	-1	1	-1	-1	-1	-1	-1	1
-1	-1	-1	-1	1	-1	1	-1	3
-1	-1	1	1	-1	-1	-1	-1	1
1	-1	1	1	1	1	1	1	5
1	-1	1	-1	-1	-1	-1	-1	3
1	-1	1	1	-1	-1	1	-1	2
1	1	1	1	-1	-1	1	1	3

1	1	1	1	1	-1	1	-1	2
1	1	1	1	-1	-1	1	-1	0
1	1	1	1	1	1	1	1	3
-1	1	1	1	1	1	1	1	2
1	-1	1	1	-1	-1	1	-1	1
1	-1	1	1	-1	-1	-1	-1	1
1	1	1	1	1	1	1	1	0
1	-1	-1	-1	-1	-1	-1	-1	2
1	-1	1	1	1	1	1	1	3
-1	-1	-1	-1	-1	-1	1	-1	0
-1	-1	1	-1	1	-1	1	-1	0
-1	-1	1	1	1	-1	1	-1	1
1	1	1	1	1	-1	1	-1	3
1	-1	1	1	-1	-1	1	-1	2
1	1	1	1	1	1	1	1	2
1	1	1	-1	1	1	1	1	1
1	-1	1	-1	1	1	1	-1	0
1	1	1	-1	-1	-1	-1	-1	0
1	1	1	1	1	1	1	1	0
1	-1	-1	-1	-1	1	1	-1	1
1	-1	1	-1	-1	-1	1	-1	0
-1	-1	-1	-1	-1	-1	-1	1	0
1	1	1	-1	-1	-1	1	-1	0
1	-1	-1	-1	-1	-1	1	-1	0
1	1	1	1	1	-1	1	-1	3
1	1	1	-1	-1	-1	1	-1	3
-1	-1	-1	1	1	-1	1	-1	3
1	-1	1	1	1	-1	1	1	2
1	-1	1	-1	1	1	-1	1	0
1	1	1	1	-1	1	1	1	8
1	-1	-1	1	1	-1	1	-1	2
-1	-1	-1	-1	-1	1	1	-1	0
-1	-1	-1	-1	-1	-1	1	1	2
1	-1	-1	1	-1	-1	-1	-1	2
1	-1	1	-1	-1	-1	-1	-1	0
1	-1	1	-1	-1	-1	1	-1	1
1	-1	-1	1	1	1	1	-1	0
1	1	1	1	1	1	1	1	2
-1	-1	-1	1	-1	-1	1	-1	0
-1	-1	-1	1	-1	-1	1	-1	1
1	1	1	-1	1	1	1	1	2
1	-1	1	1	-1	-1	1	-1	5
-1	-1	1	-1	-1	-1	1	-1	0
1	-1	1	-1	-1	1	1	-1	2

1	-1	1	1	1	-1	1	1	1
-1	-1	-1	-1	-1	-1	-1	-1	0
1	-1	1	1	1	1	-1	1	2
1	-1	-1	-1	1	1	1	-1	0
-1	-1	-1	1	1	-1	1	-1	1
1	-1	1	1	1	-1	1	-1	0
1	-1	1	1	1	1	1	1	1
1	1	1	1	1	1	1	1	10
1	-1	1	1	-1	-1	1	-1	1
-1	-1	-1	1	-1	-1	-1	-1	2
1	1	1	1	1	1	1	1	2
1	-1	1	-1	-1	-1	1	-1	2
1	1	1	1	1	-1	1	-1	1
-1	-1	1	1	1	1	1	-1	1
1	1	1	1	1	1	1	1	1
-1	-1	-1	-1	-1	-1	-1	-1	5
-1	-1	-1	1	-1	-1	1	-1	20
-1	1	1	1	1	1	1	1	0
1	-1	1	-1	1	1	1	-1	1
1	-1	1	1	1	-1	1	-1	3
1	1	1	1	-1	1	1	-1	2
1	-1	1	-1	-1	-1	1	-1	0
1	1	1	1	-1	-1	1	-1	0
-1	-1	-1	-1	-1	-1	-1	-1	0
1	1	1	1	1	1	1	1	1
1	1	1	1	1	1	1	1	1
1	-1	1	1	-1	-1	1	-1	0
-1	1	1	-1	-1	-1	1	-1	1
1	-1	1	-1	-1	-1	1	-1	1
-1	-1	1	1	1	-1	1	-1	3
1	-1	1	-1	-1	-1	1	-1	3
-1	-1	1	1	-1	-1	-1	-1	1
-1	-1	-1	1	-1	-1	1	-1	4
1	-1	-1	1	-1	-1	-1	-1	0
-1	-1	1	1	-1	-1	-1	-1	1
-1	-1	-1	-1	-1	-1	-1	-1	0
1	1	1	-1	-1	-1	-1	-1	1
1	-1	1	-1	1	1	1	-1	2
1	-1	1	1	-1	-1	1	-1	0
1	1	1	1	1	1	1	1	0
1	-1	1	1	1	-1	1	-1	10
1	1	1	1	-1	1	-1	1	0
1	1	-1	-1	1	-1	1	1	2
1	-1	1	1	1	-1	-1	-1	1

1	-1	1	1	1	1	1	-1	2
-1	-1	1	-1	-1	-1	1	-1	1
1	-1	-1	1	1	-1	-1	1	5
1	-1	1	-1	-1	-1	1	1	1
1	-1	1	-1	-1	-1	-1	-1	1
1	1	1	1	1	-1	1	1	1
1	1	1	1	1	-1	1	-1	1
1	-1	-1	-1	-1	-1	1	-1	3
-1	-1	1	1	-1	-1	1	-1	1
1	1	1	-1	-1	-1	-1	-1	10
1	-1	1	1	-1	-1	1	-1	1
1	-1	1	1	1	1	1	-1	0
1	-1	-1	-1	1	1	1	-1	3
1	1	-1	1	-1	1	1	-1	1
-1	-1	1	1	1	1	1	-1	5
1	1	1	-1	1	1	1	1	1
1	-1	1	-1	-1	-1	-1	-1	2
1	1	1	1	1	-1	1	-1	0
1	-1	-1	1	1	-1	1	-1	5
1	-1	1	-1	-1	-1	1	1	0
-1	-1	1	1	-1	-1	1	-1	1
1	1	1	1	1	1	1	1	0
1	-1	1	1	1	-1	-1	-1	3
1	-1	-1	1	-1	-1	1	-1	3
1	1	1	1	1	-1	1	-1	2
1	1	1	1	1	1	1	-1	0
1	1	1	-1	1	-1	1	1	0
-1	-1	1	1	1	-1	-1	-1	4
1	-1	1	1	1	-1	1	1	4
1	-1	1	1	-1	-1	1	-1	0
1	-1	1	1	1	-1	1	1	6
1	-1	1	1	1	1	1	1	4
-1	-1	-1	-1	-1	-1	1	-1	1
1	-1	-1	-1	-1	-1	1	-1	0
1	-1	1	1	-1	-1	1	-1	1
1	1	1	1	1	1	1	-1	1
1	1	1	1	1	-1	1	1	1
1	1	1	1	1	1	1	1	2
1	1	1	1	-1	1	1	-1	1
1	-1	1	-1	-1	-1	1	1	0
-1	-1	1	-1	-1	-1	1	1	0
1	-1	-1	-1	1	1	1	-1	0
1	1	1	1	-1	1	1	-1	0
1	1	1	1	1	1	1	1	2

-1	-1	-1	-1	-1	-1	-1	-1	1
-1	1	1	1	-1	-1	-1	-1	0
-1	-1	-1	-1	-1	-1	-1	-1	2
-1	-1	-1	-1	-1	-1	-1	-1	5
1	-1	1	1	1	1	1	-1	5
1	1	1	1	-1	-1	1	-1	1
1	1	1	-1	-1	-1	1	1	1
1	1	1	1	1	-1	1	-1	7
1	1	1	1	1	1	1	1	7
-1	1	1	-1	-1	-1	-1	-1	0
1	1	1	1	1	1	1	1	2
-1	1	-1	1	-1	1	1	1	0
-1	-1	1	1	-1	-1	-1	-1	3
-1	1	-1	-1	-1	-1	1	1	1
-1	-1	1	1	1	-1	1	-1	2
1	-1	1	1	1	-1	1	-1	2
1	-1	1	1	1	-1	1	-1	1
1	1	1	1	1	1	1	1	1
-1	-1	-1	-1	-1	-1	1	-1	3
1	-1	-1	1	-1	-1	1	1	2
1	1	1	1	1	1	1	-1	0
1	-1	1	-1	1	-1	1	-1	0
1	1	1	1	1	1	1	1	2
1	1	1	1	1	1	1	1	6
-1	-1	1	1	-1	1	1	-1	0
-1	-1	1	1	1	-1	1	-1	1
-1	-1	-1	-1	-1	-1	-1	1	5
-1	-1	-1	-1	1	-1	1	-1	0
1	1	1	1	1	1	1	1	1
1	1	1	1	1	1	1	1	1
1	1	1	1	1	1	1	1	4
1	1	1	1	-1	-1	-1	-1	3
-1	-1	-1	-1	-1	-1	-1	-1	4
1	-1	1	1	1	-1	1	-1	1
1	-1	-1	1	1	-1	1	-1	2
1	-1	1	1	1	1	1	1	1
1	-1	1	1	-1	-1	1	-1	0
1	-1	1	1	1	-1	1	-1	2
1	1	1	1	1	1	1	-1	2
-1	-1	-1	-1	-1	1	-1	-1	2
1	1	-1	1	1	-1	-1	-1	2
1	-1	1	1	1	-1	1	-1	5
-1	-1	-1	-1	-1	-1	-1	-1	0
-1	-1	-1	-1	-1	-1	1	-1	3

1	1	1	1	1	1	1	1	1
1	1	1	1	1	1	1	1	1
1	1	1	1	1	1	1	1	10
-1	-1	1	-1	-1	-1	-1	-1	4
1	-1	1	1	1	1	1	-1	0
1	1	1	1	1	1	1	1	2
1	1	1	1	1	-1	-1	-1	4
1	1	1	1	1	1	1	1	5
1	1	1	1	1	1	1	1	3
1	-1	1	1	1	-1	-1	-1	0
1	-1	-1	-1	-1	-1	1	-1	2
-1	-1	-1	-1	-1	-1	1	-1	1
1	1	1	-1	-1	-1	1	1	0
1	1	-1	-1	1	-1	1	1	2
1	-1	-1	-1	-1	-1	1	-1	1
1	1	1	-1	1	1	1	1	0
1	1	1	1	1	-1	-1	-1	1
1	1	1	1	1	-1	1	-1	4
-1	-1	-1	1	-1	-1	-1	-1	0
-1	-1	-1	-1	-1	-1	-1	-1	0
-1	-1	-1	-1	-1	-1	1	-1	1
1	-1	1	1	-1	1	1	-1	3
-1	-1	1	-1	-1	-1	1	-1	2
1	-1	1	-1	-1	-1	-1	1	8
-1	-1	-1	-1	-1	-1	-1	-1	0
1	1	1	1	1	1	1	1	0
-1	-1	1	1	-1	-1	1	-1	0
1	-1	1	-1	-1	1	1	-1	0
1	1	1	1	1	1	1	1	3
1	1	1	1	-1	1	1	-1	2
-1	-1	-1	-1	-1	-1	-1	-1	1
1	1	1	1	1	-1	-1	-1	1
-1	1	1	-1	1	-1	-1	-1	1
1	1	1	1	1	1	1	1	3
1	-1	1	1	-1	-1	1	-1	1
1	1	1	1	-1	-1	1	1	2
1	1	-1	1	-1	1	1	1	3
1	1	1	1	1	1	1	1	3
1	1	1	1	1	1	1	1	4
1	-1	1	1	-1	-1	1	-1	4
1	1	1	1	1	-1	1	-1	3
1	1	1	1	1	1	1	1	0
1	1	1	1	1	-1	1	1	4
-1	-1	-1	-1	-1	-1	-1	-1	3

1	-1	1	1	1	1	1	1	1
-1	-1	1	1	1	-1	-1	-1	0
1	-1	1	1	-1	-1	1	-1	1
1	-1	1	1	-1	-1	1	-1	1
-1	-1	1	1	-1	1	1	-1	1
1	1	1	-1	1	-1	1	1	2

es do you know? She\he has kidney failure
