# Supplementary material for: Accurate statistical methods to cover the aspects of the increase in the incidence of kidney failure: A survey study in Ha’il -Saudi Arabia
Source: PLoS One. 2024 Aug 28;19(8):e0309226. doi: 10.1371/journal.pone.0309226 (PMC11357112; doi:10.1371/journal.pone.0309226)
Supplement: Attachment — Submitted filename: Comments.docx [file pone.0309226.s002.docx]

Dear Reviewer, 1

I would like to say thank you for the very excellent comments, which add a very wonderful addition to the scientific paper. The following table shows the addition by the authors.

| Reviewer 1 comments | Authors comments |
| --- | --- |
| Related works must be presented elaboratively | Done in third paragraph from introduction section |
| Number of references needs to be increased | Done we added refences 6, 7, 8, 9 and 15 |
| Significance of the work needs to be highlighted clearly | Done in second paragraph from introduction section that: The study aims to identify the actual reasons… |
| English language needs to be rechecked and minor corrections are necessary | Done. We highlighted as follow:   - Line 3, 4, 5, 18 in abstract - Line 3, 5 , 27 in introduction - Subsection 2.3.1 step 5 : differences. - Third line in conclusion. |

Dear Reviewer, 2

I would like to say thank you for the very excellent comments, which add a very wonderful addition to the scientific paper. The following table shows the addition by the authors.

| Reviewer 2 comments | Authors comments |
| --- | --- |
| Abstract: result section: the ℎ9, ℎ8, ℎ1, ℎ6, ℎ14, ℎ18, need to be clarified for the readers. I suggest the authors should use the actual findings to be easily identified by the readers. | Done. We use the actual finding in abstract |
| body of the paper: result section: the repetition of the words [[EQUATION]], [[EQUATION]] may not be understood to the readers, can use other forms of findings, so as to be easily read by the readers? Also this is observed in the conclusion section. | Done in subsection 2.3.1 |
| The author should identify the validity of the two methods: Analysis by supersaturated designs and Analysis by design with edges. Are these methods compared to other developed methods? | Done in fourth paragraph from introduction section that The validity of the two methods…. |

Authors

Dr Talal

Dr **Dalia**
